# Effect of Human Myotubes-Derived Media on Glucose-Stimulated Insulin Secretion

**DOI:** 10.1155/2017/1328573

**Published:** 2017-02-14

**Authors:** Maria L. Mizgier, Luis R. Cataldo, Juan Gutierrez, José L. Santos, Mariana Casas, Paola Llanos, Ariel E. Contreras-Ferrat, Cedric Moro, Karim Bouzakri, Jose E. Galgani

**Affiliations:** ^1^Departamento de Nutrición, Diabetes y Metabolismo, Escuela de Medicina, Pontificia Universidad Católica de Chile, Santiago, Chile; ^2^Centro de Estudios Moleculares de la Célula, Instituto de Ciencias Biomédicas, Facultad de Medicina, Universidad de Chile, Santiago, Chile; ^3^Institute for Research in Dental Sciences, Facultad de Odontología, Universidad de Chile, Santiago, Chile; ^4^Exercise Science Laboratory, School of Kinesiology, Faculty of Medicine, Universidad Finis Terrae, Santiago, Chile; ^5^INSERM UMR1048, Institut des Maladies Métaboliques et Cardiovasculaires, Université Paul Sabatier, Toulouse, France; ^6^Departement de Génétique et Développement, CMU, Université de Genève, Genève, Switzerland; ^7^UMR DIATHEC, EA 7294, Centre Européen d'Etude du Diabète, Université de Strasbourg, Strasbourg, France; ^8^UDA-Ciencias de la Salud, Carrera de Nutrición y Dietética, Escuela de Medicina, Pontificia Universidad Católica de Chile, Santiago, Chile

## Abstract

Fasting to postprandial transition requires a tight adjustment of insulin secretion to its demand, so tissue (e.g., skeletal muscle) glucose supply is assured while hypo-/hyperglycemia are prevented. High muscle glucose disposal after meals is pivotal for adapting to increased glycemia and might drive insulin secretion through muscle-released factors (e.g., myokines). We hypothesized that insulin influences myokine secretion and then increases glucose-stimulated insulin secretion (GSIS). In conditioned media from human myotubes incubated with/without insulin (100 nmol/L) for 24 h, myokines were qualitatively and quantitatively characterized using an antibody-based array and ELISA-based technology, respectively. C57BL6/J mice islets and Wistar rat beta cells were incubated for 24 h with control and conditioned media from noninsulin- and insulin-treated myotubes prior to GSIS determination. Conditioned media from insulin-treated versus nontreated myotubes had higher RANTES but lower IL6, IL8, and MCP1 concentration. Qualitative analyses revealed that conditioned media from noninsulin- and insulin-treated myotubes expressed 32 and 23 out of 80 myokines, respectively. Islets incubated with conditioned media from noninsulin-treated myotubes had higher GSIS versus control islets (*p* < 0.05). Meanwhile, conditioned media from insulin-treated myotubes did not influence GSIS. In beta cells, GSIS was similar across conditions. In conclusion, factors being present in noninsulin-stimulated muscle cell-derived media appear to influence GSIS in mice islets.

## 1. Introduction

Regulation of insulin secretion is critical for understanding glucose homeostasis under (patho)physiological conditions. Such regulation is particularly complex in the transition from fasting to postprandial state on which its secretion must be tightly adjusted to insulin needs, so tissue glucose supply is assured while hypo- and hyperglycaemia are prevented.

Skeletal muscle plays an active role controlling circulating glucose concentration. On the one hand, this tissue is a major site of insulin-stimulated glucose disposal [1], which is crucial in the adaptation to the rapid increase in glucose flux into circulation after a meal. On the other hand, skeletal muscle might also influence insulin secretion by interacting with pancreas through humoral factors [2–6]. Interestingly, two skeletal muscle-specific genetic mice models characterized by altered glucose metabolism (a knock-out for peroxisome proliferator-activated receptor gamma coactivator 1-alpha [PGC1-alpha^−/−^] [5] and a transgenic for muscle-specific RING-finger 1 protein [Murf1] [6]) present abnormal in vivo insulin secretion. Such findings have been considered to be indicative of a putative endocrine factor mediating a muscle-pancreas crosstalk.

In humans, support for this hypothesis comes indirectly from in vivo studies aimed at assessing the effect of insulin on its secretion [7, 8]. These studies found higher glucose-stimulated insulin secretion (GSIS) following a 4 h isoglycemic-hyperinsulinemic clamp when compared with a 4 h saline infusion [7, 8]. This finding was in line with an in vitro study reporting higher insulin secretion after insulin stimulation [9]. However, most of the in vitro studies have shown that insulin inhibits its own secretion [10–12]. Thus, enhanced GSIS after insulin versus saline infusion may have an alternative explanation. We propose that the drastic increase in skeletal muscle glucose disposal after insulin (versus saline) infusion may trigger the release of a humoral factor having influence on insulin secretion [2]. In this regard, we recently highlighted an inverse, insulin sensitivity-independent association between 24 h whole-body carbohydrate oxidative disposal (an indirect marker of skeletal muscle glucose metabolism) and 24 h insulin secretion in humans [13]. Such finding may suggest that glucose disposal, particularly at the level of its oxidation, may drive insulin secretion [14]. Further support to this hypothesis underlies on the observation that skeletal muscle-specific PGC1-alpha^−/−^ versus wild-type mice had impaired in vivo but normal in vitro (isolated islets) GSIS. This observation suggests that a humoral factor is mediating an in vivo interaction between skeletal muscle and pancreas.

The notion that a muscle-pancreas crosstalk exists has been fairly accepted [15, 16], although the effect of muscle-released factors including myokines on pancreatic insulin secretion has not been proven in in vivo animal or human models. A reasonable, feasible, and first-step approach to test this hypothesis is to evaluate the effect of conditioned media from insulin- and noninsulin-treated human myotubes on GSIS from isolated pancreatic islets or beta cells. This model was used by Bouzakri et al. [3], who observed increased GSIS in rat and human primary beta cells incubated with conditioned media from nonstimulated human muscle cells.

We also included the determination of 5 proteins in the conditioned media as candidate mediators. We selected IL6, IL8/CXCL8, MCP1/CCL2, fractalkine/CX3CL1, and RANTES/CCL5, which are known to be released from muscle cells [3, 17, 18]. Some of them (IL6, fractalkine/CX3CL1, and RANTES/CCL5) have been also shown to influence in vitro insulin secretion [4, 5, 19, 20]. In turn, we recently reported that circulating IL8/CXCL8 concentration directly associates with in vivo insulin secretion in humans [21].

## 2. Methods

### 2.1. Experimental Design

Human primary myotubes were incubated for 24 h with or without 100 nmol/L insulin [22]. Then, conditioned media were collected and stored at −80°C for later analysis, which included (i) characterization of the myokine profile and (ii) incubation of pancreatic mice islet and rat beta cells to evaluate basal insulin secretion and GSIS. In parallel, insulin-induced metabolic changes at the level of glycogen content and synthesis, glucose oxidation, and extracellular lactate concentration were assessed.

### 2.2. Myotubes Differentiation

Skeletal muscle cells were obtained from* vastus lateralis* biopsies of lean healthy volunteers (4 males, 24 ± 1 years, 23 ± 1 kg/m^2^, and 85 ± 2 mg/dL fasting blood glucose). All volunteers gave written informed consent and the protocol was approved by an institutional ethics committee of the Toulouse Hospital (number 0816302). Studies were performed according to the latest version of the Declaration of Helsinki and the Current International Conference on Harmonization guidelines. The clinical study was registered at Clinicaltrials.gov NCT01083329 and EudraCT 2009-012124-85.

Myoblasts were isolated and grown as previously described [23]. Briefly, myoblasts were isolated by trypsin digestion, preplated on an uncoated Petri dish for one hour to remove fibroblasts, and subsequently transferred to T-25 collagen-coated flasks in Dulbecco's modified Eagle's medium (DMEM) low glucose (5.5 mmol/L) supplemented with 10% foetal bovine serum (FBS) and growth factors (human epidermal growth factor, BSA, dexamethasone, gentamicin, fetuin, and amphotericin B [Fungizone, Invitrogen]). Cells were pooled and grown at 37°C and 5% CO_2_. Differentiation of myoblasts into myotubes was initiated at approximately 90% confluence by switching to alpha-minimum essential medium (alpha-MEM), Glutamax™ supplement with antibiotics, 2% FBS, and fetuin. The medium was changed every other day, and cells were grown up to 5-6 days.

### 2.3. Myotube-Conditioned Media Generation

Myotubes were incubated for 24 h with/without 100 nmol/L recombinant human insulin (Sigma-Aldrich) in alpha-MEM, Glutamax supplement, without FBS. At the end of the treatment, conditioned media were collected and stored at −80°C until utilization. Once thawed on ice, conditioned media were centrifuged at 14000*g*, 10 min at 4°C to eliminate any cell debris. Cell death was assessed by chemiluminescent quantification of adenylate kinase activity (ToxiLight, Lonza Group Ltd., Basel, Switzerland) in conditioned media from noninsulin- and insulin-treated myotubes.

### 2.4. Total Glycogen Assay

After 24 h with/without 100 nmol/L insulin, total glycogen content was determined using an enzymatic method (amyloglucosidase [Sigma-Aldrich]) and the glucose amount obtained was quantified using a commercial kit (DiaSys “Glucose GOD FS”). All assays were performed in triplicate and were normalized to protein amount (BCA Protein Assay kit, Pierce).

### 2.5. Glycogen Synthesis Assay

Myotubes were preincubated with glucose- and serum-free alpha-MEM for 90 min and then exposed to DMEM supplemented with D[U-^14^C]glucose (1 *μ*Ci/mL; PerkinElmer) with/without 100 nmol/L insulin for 3 h. After incubation, glycogen synthesis was determined as described previously [22, 23]. All assays were performed in triplicate and normalized to protein amount (BCA Protein Assay kit, Pierce).

### 2.6. Glucose Oxidation Assay

Myotubes were preincubated with glucose- and serum-free alpha-MEM for 90 min. This incubation was followed by a 3-hour incubation with D[U-^14^C]glucose (1 *μ*Ci/mL) and 5.5 mmol/L of nonlabelled (cold) glucose with/without 100 nmol/L insulin. After incubation, ^14^CO_2_ was measured as previously described [22, 23]. All assays were performed in triplicate and normalized to protein amount (BCA Protein Assay kit, Pierce).

### 2.7. Extracellular Lactate Determination

Lactate was measured in myotube supernatant after 24 h treatment with/without insulin using a commercial kit (Lactate PAP, Biomérieux, France). All assays were performed in triplicate and normalized to protein amount (BCA Protein Assay kit, Pierce).

### 2.8. Myokine Concentration in Conditioned Media

In conditioned media from myotubes treated for 24 h with/without 100 nmol/L insulin, quantitative determination of 5 a priori selected cytokines/chemokines was performed by using multiplex analysis (Luminex, R&D System for IL6, IL8/CXCL8, RANTES/CCL5, and MCP1/CCL2) or ELISA (for fractalkine/CX3CL1; R&D System).

Further analysis included qualitative determination of 80 proteins through a membrane-based antibody array (C-series AAH-CYT-5, from Raybiotech®). Membranes were revealed using a blot scanner (C-Digit®, LI-COR) and spots densitometry was measured using the Image Studio™ software, following the manufacture instructions.

### 2.9. Murine Pancreatic Islets and *β* Cell Isolation

Male C57BL6/J mice were used for all mouse islets experiments and Wistar rats for all rat beta cells experiments. Animals were housed in a temperature- and light-controlled room and were allowed to consume standard chow and water ad libitum. All mice experiments were carried out in accordance with the National Research Council (NRC) Publication Guide for Care and Use of Laboratory Animals (copyright 1996, National Academy of Science) and approved by the Ethics Committee for Animal Welfare from the School of Medicine of the Pontifical Catholic University of Chile. Rat experiments were carried out according to protocols approved by the State Commissioner on Animal Care. Adult 8-week old mice and 150–200 g rats were anesthetized with a mix of ketamine: xylazine (0.18 mg : 0.012 mg per gram of animal) by intraperitoneal injection. Pancreas was perfused with collagenase (0.21 mg/mL of Liberase TL [Roche] for mice and 0.90 mg/mL of collagenase [Sigma] for rats) through the common bile duct prior to euthanasia (by incision of the chest cavity to produce a bilateral pneumothorax). After verification of death, pancreas was removed out of the animal. Islets were isolated after pancreas digestion (37°C for 14 min), followed by Histopaque® 1077 (Sigma) density gradient separation and handpicked purification. Mice islets were cultured until the next day in RPMI 1640 medium containing 11.2 mmol/L glucose, 10% FBS, 110 *μ*g/mL sodium pyruvate, and antibiotics (110 U/mL penicillin and 110 *μ*g/mL streptomycin). Rat islets were trypsinized and beta cells were purified using a fluorescence-activated cell sorter (FACS), by autofluorescence to yield a population of more than 95% beta cells. Sorted rat beta cells were washed in 10 mL sterile DMEM (GIBCO, Invitrogen) containing 11.2 mmol/L glucose, 10% FCS, 110 *μ*g/mL sodium pyruvate, and antibiotics (110 U/mL penicillin, 110 *μ*g/mL streptomycin, and 50 *μ*g/mL gentamycin). Aliquots of 3 × 10^5^ cells were seeded in nonadherent Petri dishes and incubated overnight at 37°C. The next day, cells were resuspended at a density of 4 × 10^5^ cells/mL in DMEM. Aliquots (50 *μ*L) of this suspension were plated as droplets at the center of Petri dishes previously coated with 804G extracellular matrix [24] and incubated at 37°C until the next day.

### 2.10. Murine Pancreatic Islets and Beta Cell Incubation with Myotube-Derived Conditioned Media

Mice islets (5 per condition) and rat beta cells were incubated for 24 h with (i) unconditioned (control) media; (ii) conditioned media from noninsulin-treated myotubes; (iii) conditioned media from insulin-treated myotubes; and (iv) unconditioned (control) media with 100 nmol/L recombinant insulin. All media were supplemented with 10% SBF prior to islet or beta cell incubation.

### 2.11. Glucose-Stimulated Insulin Secretion

After 24 h incubation, mice islets and rat beta cells were washed for one or two hours (resp.) by incubating with Krebs Ringer HEPES buffer (KRH in mmol/L: 137 NaCl, 4.8 KCl, 1.2 KH_2_PO_4,_ 1.2 MgSO_4,_ 2.5 CaCl_2,_ 5 NaHCO_3_, 16 HEPES, and 0.1% BSA) at 2.8 mmol/L glucose, and supernatant was eliminated. Then, mice islets and rat beta cells were incubated for one hour with KRH 2.8 mmol/L glucose (basal insulin secretion) followed by one-hour incubation at 16.7 mmol/L glucose (i.e., GSIS). All incubations were performed at 37°C and 5% CO_2_. Supernatants were collected, while islets and beta cells were lysed in HCl-Ethanol. Supernatants and lysates were stored at −20°C for insulin determination. For mice islets, insulin was determined by ELISA (Merck-Millipore) by RIA for rat beta cells. Insulin secretion is expressed as a percentage of the total content. All experiments were run in triplicate.

### 2.12. Quantitative Real-Time PCR of Protein Receptors in Mice Pancreatic Islets

Two independent pools of at least 400 islets were obtained from ~10 mice each. Islets were incubated overnight in complete RPMI 1640 medium as described above and stored at −80°C in lysis buffer with 1% 2-mercaptoethanol (Life Technologies) for later extraction and analysis. Total RNA was isolated using the PureLink™ RNA Mini Kit (Life Technologies), treated with DNase (on column PureLink DNAse, Life Technologies), and quantified with a Nanodrop 2000 spectrophotometer (Thermo Scientific). cDNA was synthesized with the AffinityScript QPCR cDNA Synthesis Kit (Agilent Technologies), using 500 ng total RNA in a 20 *μ*L reaction volume. Gene amplification was carried out using the Brilliant II SYBR® Green QRT-PCR AffinityScript kit (Agilent Technologies) on a Stratagene MX3000P thermocycler. Primers were purchased from Integrated DNA Technologies, Inc. (Table 1). Relative expression of mRNAs was determined after normalization against cyclophilin as an internal reference and calculated by the 2^−ΔΔCt^ method.

### 2.13. Statistical Analyses

Unless stated otherwise, all data are expressed as mean ± SEM of multiple experiments. All statistical comparisons were done by two-tailed* t* tests or two-way ANOVA with Tukey post hoc test, as appropriate. Values of *p* < 0.05 were considered significant. Statistical analyses were performed using GraphPad Prism 6.0 for Windows (GraphPad Software Inc., San Diego, CA).

## 3. Results

### 3.1. Myotubes Glucose Metabolism

**Figure 1:**
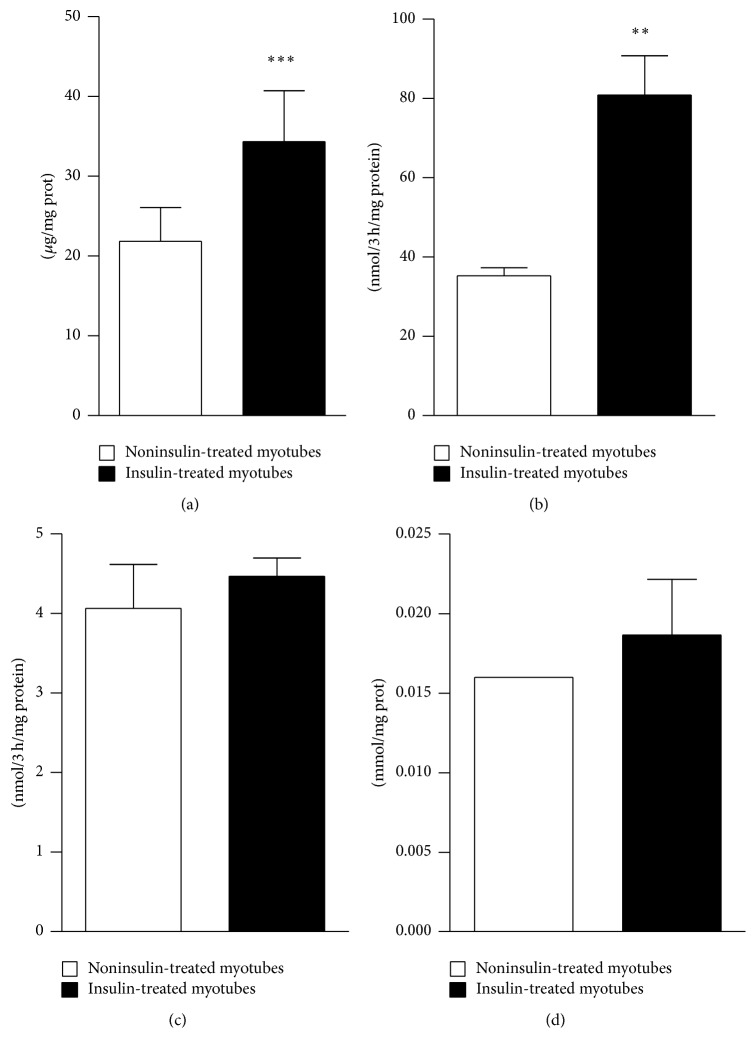
Myotubes metabolic changes in response to insulin. Glycogen content (a) and extracellular lactate content (d) were determined after 24 h with/without 100 nmol/L insulin treatment. Glycogen synthesis (b) and glucose oxidation (c) were determined after 3 h incubation with D[U-^14^C]glucose with/without 100 nmol/L insulin. Mean ± SEM. ^*∗∗*^*p* < 0.01 and ^*∗∗∗*^*p* < 0.001, two-tailed* t* test.

As expected, insulin increased myotubes glycogen content after 24 hours by 1.6 ± 0.3-fold (*p* < 0.001) and its synthesis over 3 hours by 2.3 ± 0.9-fold (*p* < 0.002) (Figures 1(a) and 1(b)). However, glucose oxidation and lactate concentration remained similar when compared with nontreated myotubes (Figures 1(c) and 1(d)).

### 3.2. Myokine Content in Conditioned Media from Human Myotubes

**Figure 2:**
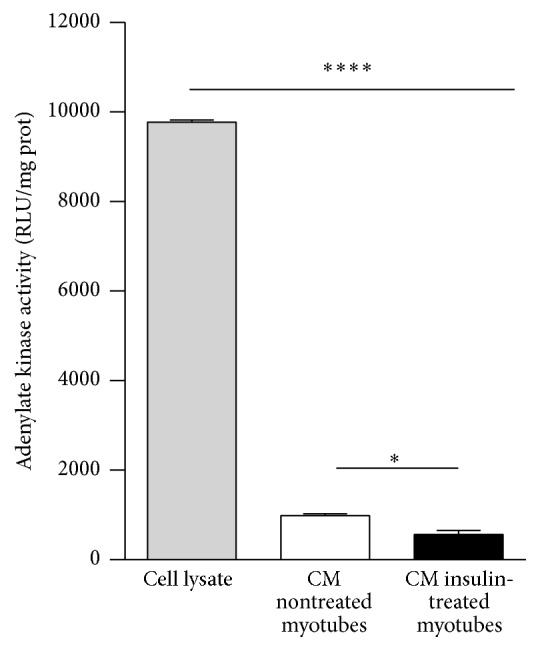
Myotube death in conditioned media from noninsulin- and insulin-treated myotubes. Cell death was assessed by chemiluminescent quantification of adenylate kinase activity normalized to total protein content. RLU, relative light units. Mean ± SEM. ^*∗*^*p* < 0.05 and ^*∗∗∗∗*^*p* < 0.0001, one-way ANOVA with Tukey post hoc test.

**Figure 3:**
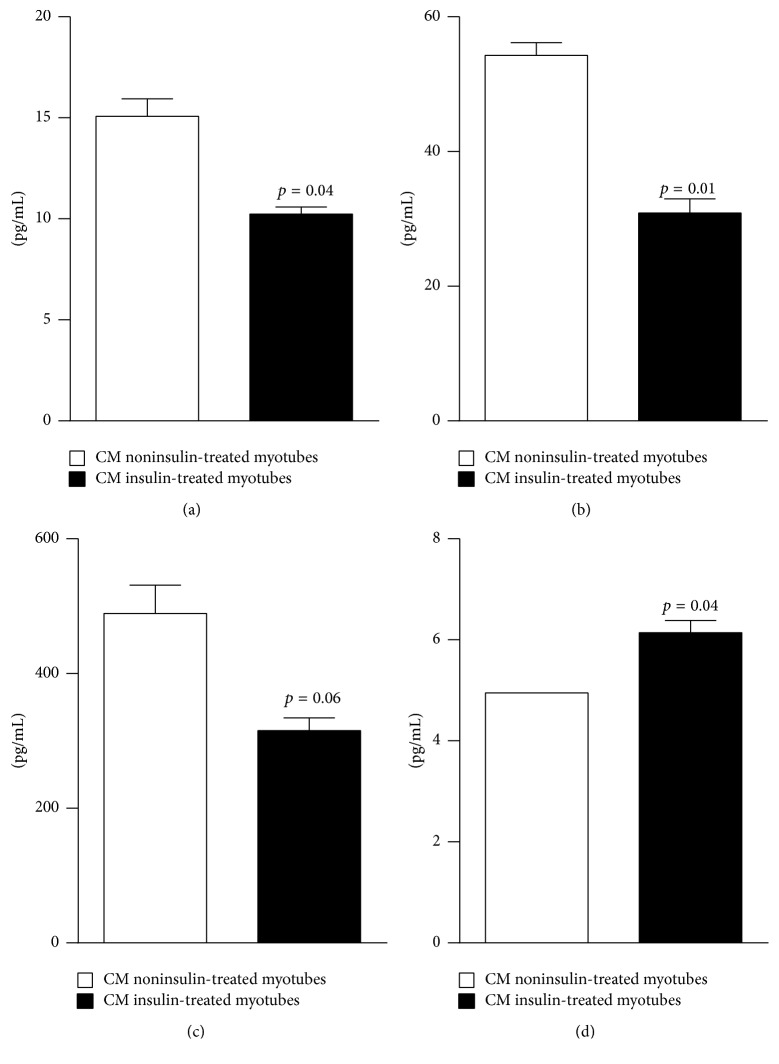
Myokine expression in conditioned media from noninsulin- and insulin-treated myotubes. IL6 (a), IL8/CXCL8 (b), MCP1/CCL2 (c), and RANTES/CCL5 (d) determined by multiplex in myotube-conditioned media from noninsulin- and insulin-treated myotubes. Mean ± SEM. Analysis by two-tailed* t*-student.

Conditioned media from noninsulin-treated and insulin-treated human myotubes showed less than 10% cell mortality relative to the cell lysate positive control (Figure 2). Four out of 5 selected myokines (IL6, IL8/CXCL8, MCP1/CCL2, and RANTES/CCL5) were found in both conditioned media (Figures 3(a)–3(d)), whereas fractalkine/CX3CL1 was not detected in any media. Conditioned media from insulin- versus noninsulin-treated myotubes had lower IL6 and IL8/CXCL8 concentration (*p* < 0.05; Figures 3(a) and 3(b)) but higher RANTES/CCL5 concentration (*p* < 0.05; Figure 3(d)). In turn, MCP1/CCL2 showed a borderline significant lower concentration in conditioned media from insulin-treated myotubes (*p* = 0.06; Figure 3(c)). Further analysis explored the presence of a larger number of proteins by using a qualitative protein array including 80 chemokines and cytokines. We detected 32 and 23 out of 80 proteins in conditioned media from noninsulin-treated and insulin-treated myotubes, respectively (see Figure S1 in Supplementary Material available online at https://doi.org/10.1155/2017/1328573). Importantly, 10 proteins that were detectable in conditioned media from noninsulin-treated myotubes became undetectable in media from insulin-treated myotubes. In turn, one protein turned detectable in response to insulin. A full list of the detected proteins before and after insulin treatment is shown in Table S1.

### 3.3. Insulin Secretion in Murine Pancreatic Islets and Beta Cells Incubated with Myotube-Derived Conditioned Media

**Figure 4:**
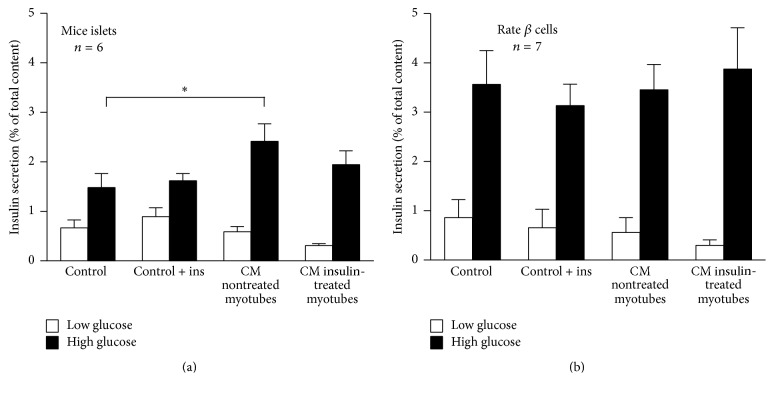
Effect of conditioned media from noninsulin- and insulin-treated myotubes on glucose-stimulated insulin secretion. Insulin secretion in isolated mice islets (a) and primary rat beta cells (b). Islets and beta cells were incubated with conditioned media from nontreated and insulin-treated myotubes for 24 h before hormone secretion assessment. Controls are unconditioned media without/with added insulin (100 nmol/L). Low glucose = 2.8 mmol/L glucose and high glucose = 16.7 mmol/L glucose. Mean ± SEM. ^*∗*^*p* < 0.05, two-way ANOVA with Tukey post hoc test.

Mice islets incubated with control media (with or without added recombinant insulin) or myotube-conditioned media (from noninsulin- and insulin-treated myotubes) had similar basal insulin secretion (Figure 4(a)). In addition, GSIS increased at similar extent in both control conditions (Figure 4(a)). However, islets incubated with noninsulin-treated myotube-conditioned media showed higher GSIS versus control (2.4 ± 0.4 vs. 1.5 ± 0.3%; *p* < 0.05. Figure 4(a)). In turn, islets incubated with conditioned media from insulin-treated myotubes had similar GSIS versus its respective control (*p* = 0.71) and also when compared with GSIS induced by conditioned media from noninsulin-treated myotubes (*p* = 0.43) (Figure 4(a)). In rat primary beta cells, insulin secretion (both at low and high glucose concentration) was not different across conditions (*p* = 0.71) (Figure 4(b)).

### 3.4. Myokine Receptors mRNA Expression in Isolated Mice Islets

**Figure 5:**
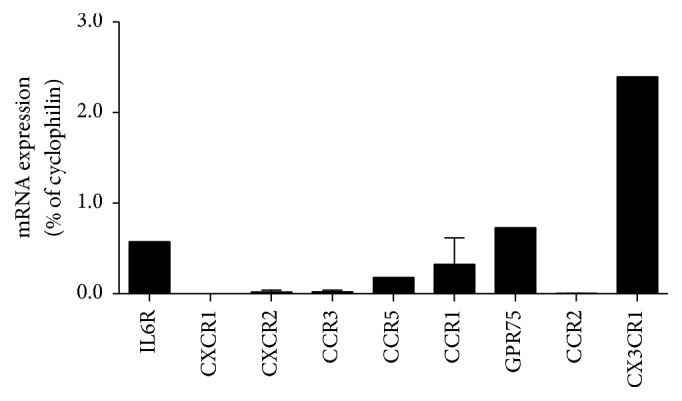
mRNA expression of myokine receptors in mouse islets. Quantification of myokine receptors mRNA in ~400 pooled islets expressed as a percentage of cyclophilin mRNA levels in the same samples. Mean ± SEM. *n* = 2.

Gene expression of selected myokine receptors was determined in isolated mice islets (receptor's and their ligands are listed in Table 2). Three out of 4 RANTES/CCL5 receptors were found in islets. Among them, GPR75 had the highest expression level followed by CCR1 and CCR5. In turn, IL6 and fractalkine/CX3CL1 receptors (IL6Ra and CX3CR1, resp.) were also expressed. Among all protein receptors measured, CXC3R1 showed the highest relative expression, whereas the common receptor for IL8/CXCL8, GRO/CXCL1, 2, and 3, NAP2/CXCL7 and ENA78/CXCL5 (CXCR1, CXCR2), MCP1/CCL2 (CCR2), and RANTES/CCL5 (CCR3) receptors did not have detectable expression (Figure 5).

## 4. Discussion

We found that human myotubes-derived media increased GSIS in isolated mice islets, whereas such effect was not observed in rat primary beta cells. In turn, conditioned media of insulin-treated myotubes did not change GSIS, both in isolated pancreatic islets and beta cells, when compared with control condition. Previously, Bouzakri et al. [3] detected increased GSIS in primary human and rat beta cells incubated with conditioned media from human myotubes. Those findings are consistent with our observation in isolated islets; however, we did not confirm that observation using primary rat beta cells. Eventually, different incubation times between studies (24 h in the present study versus 48 h) play a role. Alternatively, a potential interaction between beta and nonbeta cells may underlie the contrasting outcome between islets and beta cells. In this regard, Ellingsgaard et al. reported that IL6 enhanced GSIS in islets through increased alpha cell GLP1 secretion [4].

Our hypothesis that skeletal muscle interacts with pancreas regulating insulin secretion is mostly grounded on the classical inverse association between insulin sensitivity and its secretion [2]. Several factors found in conditioned media from muscle cells, including proteins (i.e., myokines), metabolites (e.g., lactate), and also vesicular-like structures (e.g., exosomes) [16], may mediate such putative muscle-pancreas crosstalk. Here, we focused on the presence of myokines in conditioned media.

Human myotubes conditioned media expressed 32 out of 80 detectable proteins. Apparently, none of the most highly expressed myokines (GRO/CXCL1, 2, and 3, MCP1/CCL2, IL8/CXCL8, TIMP1, TIMP2, NAP2/CXCL7, and ENA-78/CXCL5) according to our qualitative approach seemed to play a role in insulin secretion, considering that their receptors in isolated pancreatic islets showed undetectable mRNA expression under cultured conditions (i.e., RPMI 1640 at 11.2 mmol/l glucose). Certainly, alternative myokines as well as nonprotein factors may underlie our finding.

In this regard, a nonprotein factor representing a new cell-to-cell communication mode comes from exosomes. These are microvesicles carrying molecules such as microRNA that can reach distant organs and exert a (patho)physiological effect. Indeed, a recent study showed that skeletal muscle cells-derived exosomes when injected to mice targeted beta cells [15].

Additional interest was focused on the role of insulin on myokine secretion and then the effect of conditioned media from insulin-treated myotubes on GSIS. As observed for other conditions (e.g., muscle contraction-induced glycogen depletion increases muscle IL6 secretion [25, 26]), the secretion of some myokines might be sensitive to changes in insulin-dependent muscle glucose metabolism. By using a widely accepted in vitro insulin concentration (100 nmol/l) to induce muscle cell glucose metabolism [22, 27–29], we found differences in three of the studied proteins expression in conditioned media from noninsulin- versus insulin-treated myotubes (IL6, IL8/CXCL8, and RANTES/CCL5).

Thus, we observed that an increase in glycogen synthesis/content was accompanied by reduced IL6 concentration in conditioned media of insulin-treated human myotubes. Eventually, IL6 and additional myokines may mediate the well-known inverse association between insulin sensitivity and its secretion [22, 25–28]. However, such distinct myokine pattern apparently did not lead to differential GSIS from isolated pancreatic or beta cells incubated with media from insulin-treated versus control myotubes.

In part, insulin induced only minor changes in myokine secretion and GSIS from islets incubated with nonconditioned media and conditioned media from insulin-treated myotubes was similar. It cannot be ruled out that 24 h insulin exposure (100 nmol/L) may have impaired muscle cell insulin sensitivity and altered any insulin-dependent difference in the pattern of myokine secretion. Thus, some factors might be mostly released during the period of preserved insulin sensitivity, and then their release will be altered as insulin resistance develops. Even so, those factors should still be found in the media independent of the change over time in their secretion pattern. It is also possible that the myokine concentration at which islets were exposed may have been insufficient to influence GSIS. Furthermore, our model based on different species (human myotube-conditioned media and murine islets/beta cells) may add complexity to interpretation. Still, an earlier study found comparable results when rat and human beta cells were treated with conditioned media from human myotubes [3]. Finally, our in vitro model might not successfully emulate in vivo conditions on which insulin (directly or indirectly) appears to stimulate insulin secretion [8, 30]. Although such effect has been considered to be in line with in vitro evidence indicating that insulin directly enhances its own secretion [9], it must be considered that most in vitro studies found that insulin inhibits its secretion [10–12]. Thus, an alternative explanation based on humoral factors coming from insulin-sensitive tissues including skeletal muscle becomes appealing.

Taken together, these findings support the hypothesis that skeletal muscle-released factors can influence insulin secretion, which encourages the quest for identifying the nature of such factor as well as its potential in vivo role on glucose homeostasis. Any eventual physiological relevance of our findings appears independent of insulin and its action in muscle, at least under the context of our study. Thus, a mechanism mediating the interaction between insulin sensitivity and its secretion remains uncovered.

## Supplementary Material

The myokines identified in those media are listed in Table S1.
The myokines present in human myotube-derived conditioned media were assessed using an antibody-based array able to detect 80 proteins (Figure S1).

## Figures and Tables

**Table 1:** Sequences of forward and reverse primers used for PCR analyses.

Gene	Forward (5′ → 3′)	Reverse (5′ → 3′)	Product length (bp)
*Cxcr2*	atccaccttgaattctcccatc	gcctcactttcttccagttca	145
*Il6ra*	cctctgacttccatttctgct	caagaatcctcgtccatgtcc	118
*Cxcr1*	tcccgtgatatttccaaattctttc	tcccgcacacaaggaac	120
*Ccr3*	ggtgcccactcatattcatagg	ctactggactcataaaggacttagc	125
*Ccr5*	gtgctgacataccataatcgatg	tgtcttcatgttagatttgtacagc	147
*Ccr1*	aggaactggtcaggaataatagc	caaaggcccagaaacaaagtc	125
*Gpr75*	tcaggatctcagctcacaga	agatagggtcactactgcga	102
*Ccr2*	actgaggtaacatattattgtcttcca	gagccatacctgtaaatgcca	148
*Cx3cr1*	cacaatgtcgcccaaataacag	tcccttcccatctgctca	112
*Cyclophilin*	tggagagcaccaagacagaca	tgccggagtcgacaatgat	66

**Table 2:** Cytokines/chemokines receptors name and its ligands.

Receptor name	Ligand
IL6R	IL6
CXCR1	IL8/CXCL8 GRO/CXCL1,2&3 NAP2/CXCL7
CXCR2	IL8/CXCL8 GRO/CXCL1,2&3 NAP2/CXCL7ENA78/CXCL5
CCR3	RANTES/CCL5
CCR5	RANTES/CCL5
CCR1	RANTES/CCL5
GPR75	RANTES/CCL5
CCR2	MCP1/CCL2
CX3CR1	Fractalkine/CX3CL1
