# Supplementary material for: Effect of Human Myotubes-Derived Media on Glucose-Stimulated Insulin Secretion
Source: J Diabetes Res. 2017 Feb 14;2017:1328573. doi: 10.1155/2017/1328573 (PMC5329672; doi:10.1155/2017/1328573)

Supplementary legends

Table S1. Myokine presence in conditioned media from human myotubes.

Protein expression normalized to internal positive control.

Non-detectable: protein expression below 3-fold negative control.

Treated-/non-treated ratio corresponds to protein expression for non-insulin-treated relative to insulin-treated myotubes. Ratios ≤0.8 and ≥1.2 where considered as an insulin induced change.

n=2

Figure S1. Cytokines/chemokines antibody array

Cytokine/Chemokine array layout (A). Array membranes from non-insulin treated and insulin-treated myotubes conditioned media (B) of one representative experiment.

Supplementary data

Table S1

	Expression in conditioned media from		Treated-/non-treated ratio
	Non-treated-myotubes (mean ± SD)	Insulin-treated myotubes (mean ± SD)	
GCP2	0.21 ± 0.05	Non-detectable	
MIP3alpha	0.23 ± 0.06	Non-detectable	
MIF	0.25 ± 0.01	0.16 ± 0.09	0.62
CCL23	0.15 ± 0.04	Non-detectable	
ENA-78/CXCL5	0.32 ± 0.16	0.21 ± 0.19	0.64
NT3	0.29 ± 0.08	0.19 ± 0.06	0.66
MCP1/CCL2	0.87 ± 0.47	0.58 ± 0.53	0.66
PLGF	0.19 ± 0.01	Non-detectable	
GDNF	0.29 ± 0.06	0.20 ± 0.13	0.68
IL6	0.15 ± 0.07	Non-detectable	
IL16	0.16 ± 0.04	Non-detectable	
IP10	0.15 ± 0.07	Non-detectable	

HGF	0.23 ± 0.06	0.17 ± 0.11	0.74
TGFbeta2	0.28 ± 0.01	0.21 ± 0.09	0.75
FGF9	0.16 ± 0.00	Non-detectable	
IL8/CXCL8	0.87 ± 0.57	0.66 ± 0.40	0.75
TIMP2	0.54 ± 0.05	0.42 ± 0.11	0.79
M-CSF	0.17 ± 0.08	Non-detectable	
RANTES/CCL5	0.28 ± 0.05	0.23 ± 0.08	0.84
IGFBP1	0.22 ± 0.08	0.18 ± 0.11	0.84
IL1alpha	0.21 ± 0.01	0.18 ± 0.02	0.85
NAP2/CXCL7	0.43 ± 0.02	0.37 ± 0.13	0.86
IL1beta	0.18 ± 0.03	0.16 ± 0.00	0.89
TIMP1	0.57 ± 0.06	0.52 ± 0.20	0.91
OSM	0.18 ± 0.04	0.16 ± 0.07	0.91
GRO/ CXCL1,2&3	1.00 ± 0.52	0.92 ± 0.35	0.92
IL3	0.16 ± 0.05	Non-detectable	

IGFBP2	0.24 ± 0.04	0.24 ± 0.09	0.98
LIF	0.18 ± 0.00	0.18 ± 0.06	1.00
EOTAXIN2	0.16 ± 0.07	0.18 ± 0.11	1.09
IL10	0.16 ± 0.08	0.20 ± 0.05	1.22
ANG	Non-detectable	0.20 ± 0.08	
MIP-1	0.19 ± 0.06	0.50 ± 0.40	2.63

Figure S1

A

	A	B	C	D	E	F	G	H	I	J	K
1	POS	POS	POS	POS	NEG	NEG	ENA-78/ CXCL5	G-CSF	GM-CSF	GRO	GROα
2	I-309	IL1α	IL1β	IL2	IL3	IL4	IL5	IL6	IL7	IL8/ CXCL8	IL10
3	IL12p40/ p70	IL13	IL15	IFNγ	MCP1/ CCL2	MCP2/ CCL8	MCP3/ CCL7	M-CSF	MDC	MIG	MIP-1
4	MIP-1δ	RANTES/ CCL5	SCF	SDF1	TARC	TGFβ1	TNFα	TNFβ	EFG	IGF-1	ANG
5	OSM	THPO	VEGF	PDGF BB	LEPTIN	BDNF	BLC	CCL23	Eotaxin 1	Eotaxin 2	Eotaxin 3
6	FGF4	FGF6	FGF7	FGF9	Flt3 Ligand	Fractalkine/ CX3CL1	GCP2	GDNF	HGF	IGFBP1	IGFBP2
7	IGFBP3	IGFBP4	IL16	IP10	LIF	LIGHT	MCP4/ CCL13	MIF	MIP3α	NAP2/ CXCL7	NT3
8	NT4	OPN	OPG	PARC	PLGF	TGFβ2	TGFβ3	TIMP1	TIMP2	POS	POS

B

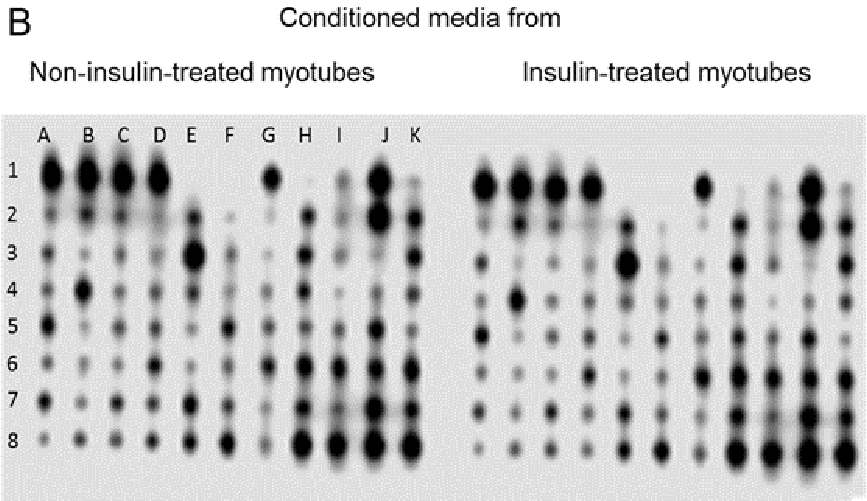

Supplement: Supplementary file 1 — The myokines identified in those media are listed in Table S1. The myokines present in human myotube-derived conditioned media were assessed using an antibody-based array able to detect 80 proteins (Figure S1). [file 1328573.f1.pdf]
